# Association of congenitally missing teeth with adult temporomandibular disorders in the urban health checkup population

**DOI:** 10.1186/s12903-023-02855-w

**Published:** 2023-03-30

**Authors:** Yundong Liu, Tao Yin, Mi He, Changyun Fang, Shifang Peng

**Affiliations:** 1 Health Management Center, Xiangya Hospital, Central South University, 87 Xiangya Road, 410008 Changsha, Hunan P.R. China; 2 Changsha Health Vocational College, 410605 Changsha, Hunan P.R. China; 3 Department of Stomatology, Xiangya Hospital, Central South University, 410008 Changsha, Hunan P.R. China; 4 Department of Infectious Diseases, Xiangya Hospital, Central South University, 87 Xiangya Road, 410008 Changsha, Hunan P.R. China

**Keywords:** Congenitally missing teeth, Number of dental quadrants with missing teeth, Temporomandibular disorders (TMD), Intra-articular TMD, Pain-related TMD

## Abstract

**Background:**

Congenitally missing tooth is the most common dental abnormality which leaves spaces in the arch, leads to numerous forms of malocclusion due to the Bolton index discrepancy and is even associated with abnormal craniofacial morphology. Even though the roles of malocclusion and tooth loss in temporomandibular disorders (TMD) development remain controversial, basic researches have found some common molecules are involved in osteoarthritis and dental agenesis. However, the association of congenitally missing teeth with TMD is unknown. We hence investigated the association of congenitally missing teeth with TMD.

**Methods:**

A cross-sectional analysis of 586 control participants (male: 287, female: 299, 38.33 ± 11.65 years) and 583 participants with non-third molar congenitally missing teeth (male: 238, female: 345, 39.13 ± 11.67 years) who consecutively received routine dental and TMD checkup according to Diagnostic Criteria for Temporomandibular Disorders Axis I in Health Management Center, Xiangya Hospital was performed. Logistic regression analysis was used to study the association of congenitally missing teeth with TMD.

**Results:**

The congenitally missing teeth group included 581 hypodontia and 2 oligodontia participants. The congenitally missing anterior teeth participants, the congenitally missing posterior teeth participants and participants with both congenitally missing anterior and posterior teeth accounted for 88.34%, 8.40% and 3.26% of the congenitally missing teeth group respectively. Congenitally missing teeth group had greater ratios of females and orthodontic history. Participants with congenitally missing teeth had a significantly higher prevalence of overall TMD (67.24%) in comparison to control participants (45.90%). After adjusting age, gender, presence of congenitally missing teeth, number of congenitally missing teeth, number of non-congenitally missing teeth, number of dental quadrants with missing teeth, visible third molar and orthodontic history, the variables of age, gender, presence of congenitally missing teeth and number of dental quadrants with missing teeth were significant for overall TMD. Multivariable logistic regression analysis showed congenitally missing tooth was significantly related with overall TMD [odds ratio (OR):1.689(1.080–2.642), *P* = 0.022], intra-articular TMD [OR: 1.711(1.103–2.656), *P* = 0.017] and pain-related TMD [OR: 3.093(1.321–7.239), *P* = 0.009].

**Conclusion:**

Congenitally missing tooth is a risk factor for TMD. When treating the congenitally missing teeth population, TMJ evaluation and multidisciplinary strategies are necessary.

## Introduction

Congenitally missing tooth is the most common dental abnormality [1–3], which leaves spaces in the arch, leads to numerous forms of malocclusion due to the Bolton index discrepancy and is even associated with abnormal craniofacial morphology [4–8]. Congenitally missing teeth could be classified into hypodontia with 1–5 congenitally missing teeth and oligodontia with ≥ 6 congenitally missing teeth. The prevalence rate and the location of the congenitally missing teeth vary to some degree all around the world [1–3]. Epidemiological investigations in the north and south Chinese general and orthodontic population [9, 10] have shown that the prevalence rate of congenitally missing teeth is about 5.89–7.48%, female population has a greater prevalence rate of congenitally missing teeth and the most common form of congenitally missing teeth is hypodontia in the mandibular incisor area. The prevalence pattern in China is different from Japanese and western population in which the most congenitally absent teeth are premolars and maxillary lateral incisors [2, 11]. Molecular researches have provided numerous genetical evidences involved in the formation of congenitally missing teeth [12]. Recently osteogenesis imperfecta patients were found to have greater prevalence rates of congenitally missing teeth [13], suggesting the shared mechanism of tooth formation and bone development. Further congenitally missing teeth are associated with not only craniofacial abnormity but also systemic diseases. Many reports have stated the association of congenitally missing teeth with malignant tumor development, indicating the common molecular mechanism [14].

Cephalometry of congenitally missing teeth population has shown Japanese hypodontia patients [7] have significantly smaller U1 to FH plane angle and A-B plane angle while in Southern Chinese subjects, hypodontia is associated with a shorter face, a flatter mandibular plane, a more pronounced chin, and a Class III skeletal profile [15]. Congenitally missing teeth are related with numerous malocclusion changes, such as deep bite and deep overjet in congenitally missing mandibular incisor patients, crossbite in congenitally missing maxillary lateral incisor and canine patients and poor intercuspal occlusion or asymmetrical malocclusion in congenitally missing premolar patients; however, the systemic description of the association between congenitally missing teeth and malocclusion is limited. Temporomandibular disorders (TMD) are associated with multiple causal factors while the roles of malocclusion and tooth loss in TMD development remain controversial [16–20]. Pullinger has found anterior open bite, unilateral maxillary lingual crossbite, large overjet, > 5–6 missing posterior teeth and RCP-ICP (retruded contact position to intercuspal contact position） slides > 2 mm increase TMD risk [21]. Uhac has shown patients with more tooth loss have temporomandibular joint (TMJ) sounds [18]. However, most epidemiological studies failed to show clinically significant differences of TMD signs and symptoms between individuals with dental arches shortened 3 to 5 occlusal units and those with complete dental arches [22]. Interestingly, Wang found when the number of missing teeth was smaller but the number of dental quadrants with missing posterior teeth was greater, TMD risk increased [23, 24]. Further basic studies have found many molecules associated with the chondrocyte and bone development are involved in TMJ osteoarthritis [25] and dental agenesis is commonly seen in osteogenesis imperfecta patients [13]. However, the association of congenitally missing teeth with TMD is unknown.

In this study, we hypothesized that congenitally missing teeth were associated with TMD in the 20–60 years adult urban health checkup population. We aimed to investigate the quantitative association of congenitally missing teeth with TMD.

## Materials and methods

### Subjects and data collection

The cross-sectional study was performed in Health Management Center, Xiangya Hospital, Central South University in Changsha city, Hunan province. The congenitally missing teeth participants aged 20–60 years received routine medical, dental and TMD checkup consecutively from 2022-03-03 to 2022-07-25. For congenitally missing teeth group, there were one or more non-third molar congenitally missing teeth which were confirmed by X-ray films and dental history. Participants with one or more congenitally missing teeth were also excluded when the condition of the unreplaced deciduous teeth was good. The control participants aged 20–60 years received routine medical, dental and TMD checkup consecutively from 2022-07-15 to 2022-07-25. For control group, there was none of non-third molar congenitally missing teeth. None of the participants had any history of oral facial trauma or rheumatoid arthritis. TMD and dental exam was completed by the first author with 10 years of experience in TMD and orofacial pain management. Clinical exam followed the Diagnostic Criteria for Temporomandibular Disorders Examination Form (DC/TMD) Axis I [26] and Wieckiewicz’s protocol [27]. The diagnosis of intra-articular TMD included: disc displacement with reduction; disc displacement with reduction with intermittent locking; disc displacement without reduction with limited opening; disc displacement without reduction without limited opening; degenerative joint disease; and dislocation. The diagnosis of pain-related TMD included: myalgia, myofascial pain with referral, arthralgia and headaches attributed to TMD. Visible third molar meant presence of clinically visible third molar. The number of non-third congenitally missing teeth and the number of non-congenitally missing teeth excluding third molar and orthodontically extracted teeth were respectively recorded. The total number of dental quadrants with congenitally and non-congenitally non-third missing teeth was also recorded.

A total of 583 participants with congenitally missing teeth and 586 control participants was included in this study. Written informed consents were obtained for all participants. The participant’s name was omitted and replaced by specific ID and the data could be easily retrieved from the specially designed database software. The study was approved by the hospital Ethics Committee (ID: 202103734) and all methods were carried out in accordance with relevant guidelines and regulations.

### Statistics

All data were presented as mean ± standard deviations for continuous variables and percentages for categorical variables. The comparison of basic clinical characteristics between the control and the congenitally missing teeth group was performed using unpaired student’s t test or non-parametric test for continuous variables and Chi Square test for categorical variables as appropriate. Logistic regression analysis was used to study the association of congenitally missing teeth with overall TMD, intra-articular TMD and pain-related TMD respectively. The software SPSS 21.0 (SPSS Inc., Chicago, IL, United States) was used for statistical analysis. *P* < 0.05 was considered as statistically significant.

## Results

### Basic clinical characteristics

Table 1 showed the basic clinical characteristics. The control group included 287 male and 299 female participants with a mean age of 38.33 ± 11.65 years. The congenitally missing teeth group included 238 male and 345 female participants with a mean age of 39.13 ± 11.67 years. The congenitally missing teeth group included 581 hypodontia and 2 oligodontia participants. The congenitally missing anterior teeth participants, the congenitally missing posterior teeth participants and participants with both congenitally missing anterior and posterior teeth accounted for 88.34%, 8.40% and 3.26% of the congenitally missing teeth group respectively. Compared with the control group, the congenitally missing teeth group had a significantly higher level of female participants (59.18%) but a significantly lower level of visible third molar, indicating congenitally missing teeth was more common in female population and the absence of visible third molar was more common in congenitally missing teeth group. Further the percentage of orthodontic history in congenitally missing teeth population was significantly greater than the control group (Table 1).

**Table 1:** Basic clinical characteristics

Variables	Control (n = 586)	Congenitally missing teeth (n = 583)	*P* value
Age (years)	38.33 ± 11.65	39.13 ± 11.67	0.242
Gender [n (%)]	Male: 287 (48.98) Female: 299 (51.02)	Male: 238 (40.82) Female: 345 (59.18)	0.005
Number of congenitally missing teeth [n (%)]		1: 373 (63.98) 2: 179 (30.70) 3: 18 (3.09) 4: 10 (1.72) 5: 1 (0.17) 6: 2 (0.34)	
Location of congenitally missing teeth [n (%)]		Anterior: 515 (88.34) Posterior: 49 (8.40) With both anterior and posterior: 19 (3.26)	
Non-congenitally missing teeth [n (%)]	62 (10.58)	60 (10.29)	0.872
Visible third molar [n (%)]	332 (56.66)	285 (48.89)	0.008
Orthodontic history [n (%)]	20 (3.41)	53 (9.09)	< 0.001
Overall TMD [n (%)]	269 (45.90)	392 (67.24)	< 0.001
Intra-articular TMD [n (%)]	269 (45.90)	383 (65.69)	< 0.001
Pain-related TMD [n (%)]	13 (2.22)	55 (9.43)	< 0.001

The congenitally missing teeth population had a significantly higher prevalence of overall TMD (67.24%) compared to the control group (45.90%) (Table 1). Compared with the control group, the prevalence rates of intra-articular TMD and pain-related TMD were significantly higher in the congenitally missing teeth group (Table 1).

The overall TMD prevalence rate in oligodontia group was 100% and the overall TMD prevalence rate in hypodontia group was 67.13% (Table 2). The difference could not be compared due to only 2 oligodontia participants. According to the location of congenitally missing teeth, all the three groups including the congenitally missing anterior teeth group, the congenitally missing posterior teeth group and the group with both congenitally missing anterior and posterior teeth had significantly higher prevalence rates of overall TMD compared to the control group. The overall TMD prevalence rate in the group with both congenitally missing anterior and posterior teeth was greatest (78.95%). The overall TMD prevalence rate in the congenitally missing posterior teeth group was the next (73.47%). The overall TMD prevalence rate in the congenitally missing anterior teeth group was lowest (66.21%) for the congenitally missing teeth group but was still significantly greater than the control group (Table 2). The differences of the overall TMD prevalence among the three congenitally missing teeth groups did not reach statistical significance due to the low sample numbers of the congenitally missing posterior teeth group and the group with both congenitally missing anterior and posterior teeth (Table 2).

**Table 2:** Overall TMD prevalence rate comparison according to hypodontia/oligodontia and the location of congenitally missing teeth

	Groups	Overall TMD prevalence rate	*P* value (†)
Hypodontia/Oligodontia	Hypodontia	67.13% (n = 581)	< 0.001
	Oligodontia	100% (n = 2)	
	Control	45.90% (n = 586)	
Location of congenitally missing teeth	Anterior	66.21% (n = 515)	< 0.001
	Posterior	73.47% (n = 49)	< 0.001
	With both anterior and posterior congenitally missing teeth	78.95% (n = 19)	0.002
	Control	45.90% (n = 586)	

### Association of congenitally missing teeth with overall TMD

As for the overall TMD, the 6 variables of age, gender, congenitally missing teeth, number of congenitally missing teeth, number of dental quadrants with missing teeth and orthodontic history were significant by univariate analysis (Table 3). In the model adjusted for age, gender, congenitally missing teeth, number of congenitally missing teeth, number of non-congenitally missing teeth, number of dental quadrants with missing teeth, visible third molar and orthodontic history, the 4 variables of age, gender, presence of congenitally missing teeth and number of dental quadrants with missing teeth were still significant for overall TMD. The odds ratio (OR) and 95% confidence intervals (CI) of presence of congenitally missing teeth was 1.689 (1.080–2.642) (*P* = 0.022) for overall TMD in the adjusted model (Table 3).

**Table 3:** Logistic regression analysis on the association of congenitally missing teeth with overall TMD

Analysis level	Variable	*P* value	OR and 95%CI
Simple logistic regression	Age	0.020	0.988 (0.978–0.998)
	Gender	< 0.001	1.832 (1.449–2.316)
	Congenitally missing teeth	< 0.001	2.419 (1.908–3.066)
	Number of congenitally missing teeth	< 0.001	1.651 (1.421–1.917)
	Number of non-congenitally missing teeth	0.147	0.907 (0.796–1.035)
	Number of dental quadrants with missing teeth	< 0.001	1.474 (1.290–1.684)
	Visible third molar	0.294	0.883 (0.700-1.114)
	Orthodontic history	0.002	2.290 (1.339–3.914)
Multivariable logistic regression	Age	0.011	0.986 (0.975–0.997)
	Gender	< 0.001	1.732 (1.357–2.210)
	Congenitally missing teeth	0.022	1.689 (1.080–2.642)
	Number of congenitally missing teeth	0.231	0.714 (0.412–1.239)
	Number of non-congenitally missing teeth	0.110	0.708 (0.464–1.081)
	Number of dental quadrants with missing teeth	0.049	1.801 (1.003–3.235)
	Visible third molar	0.989	1.002 (0.785–1.278)
	Orthodontic history	0.159	1.496 (0.854–2.622)

### Association of congenitally missing teeth with intra-articular TMD

The 6 variables of age, gender, congenitally missing teeth, number of congenitally missing teeth, number of dental quadrants with missing teeth and orthodontic history were significant for intra-articular TMD by univariate analysis (Table 4). In the fully adjusted model, the 3 variables of age, gender and presence of congenitally missing teeth were significant for intra-articular TMD. The OR and 95% CI of presence of congenitally missing teeth was 1.711 (1.103–2.656) (*P* = 0.017) for intra-articular TMD in the adjusted model (Table 4).

**Table 4:** Logistic regression analysis on the association of congenitally missing teeth with intra-articular TMD

Analysis level	Variable	*P* value	OR and 95%CI
Simple logistic regression	Age	0.024	0.989 (0.979–0.998)
	Gender	< 0.001	1.854 (1.467–2.343)
	Congenitally missing teeth	< 0.001	2.257 (1.783–2.857)
	Number of congenitally missing teeth	< 0.001	1.557 (1.345–1.803)
	Number of non-congenitally missing teeth	0.174	0.914 (0.804–1.040)
	Number of dental quadrants with missing teeth	< 0.001	1.415 (1.241–1.613)
	Visible third molar	0.338	0.893 (0.709–1.126)
	Orthodontic history	0.002	2.367 (1.385–4.046)
Multivariable logistic regression	Age	0.015	0.987 (0.976–0.997)
	Gender	< 0.001	1.750 (1.374–2.231)
	Congenitally missing teeth	0.017	1.711 (1.103–2.656)
	Number of congenitally missing teeth	0.213	0.718 (0.426–1.210)
	Number of non-congenitally missing teeth	0.142	0.749 (0.510–1.101)
	Number of dental quadrants with missing teeth	0.064	1.68 (0.970–2.910)
	Visible third molar	0.940	1.009 (0.792–1.286)
	Orthodontic history	0.107	1.584 (0.906–2.772)

### Association of congenitally missing teeth with pain-related TMD

The 4 variables of gender, congenitally missing teeth, number of congenitally missing teeth and number of dental quadrants with missing teeth were significant for pain-related TMD by univariate analysis (Table 5). After adjustment presence of congenitally missing teeth was still significant for pain-related TMD [OR: 3.093 (1.321–7.239), *P* = 0.009] (Table 5).

**Table 5:** Logistic regression analysis on the association of congenitally missing teeth with pain-related TMD

Analysis level	Variable	*P* value	OR and 95%CI
Simple logistic regression	Age	0.101	0.982 (0.961–1.004)
	Gender	0.034	1.759 (1.044–2.963)
	Congenitally missing teeth	< 0.001	4.591 (2.480–8.500)
	Number of congenitally missing teeth	< 0.001	1.738 (1.391–2.171)
	Number of non-congenitally missing teeth	0.880	0.980 (0.751–1.278)
	Number of dental quadrants with missing teeth	< 0.001	1.678 (1.346–2.093)
	Visible third molar	0.050	0.608 (0.370-1.000)
	Orthodontic history	0.058	2.125 (0.975–4.632)
Multivariable logistic regression	Age	0.069	0.979 (0.956–1.002)
	Gender	0.104	1.562 (0.912–2.674)
	Congenitally missing teeth	0.009	3.093 (1.321–7.239)
	Number of congenitally missing teeth	0.516	0.727 (0.277–1.907)
	Number of non-congenitally missing teeth	0.607	0.817 (0.379–1.762)
	Number of dental quadrants with missing teeth	0.269	1.791 (0.637–5.034)
	Visible third molar	0.216	0.724 (0.435–1.207)
	Orthodontic history	0.592	1.251 (0.551–2.840)

## Discussion

In this study, we found congenitally missing tooth was significantly associated with TMD. The hypothesis is accepted.

TMD is caused by multiple factors. The prevalence of overall TMD and intra-articular TMD in the control group is similar to the urban Polish population [27], even though the prevalence of TMD is higher in this urban health checkup control population than in the Chinse medical student population [28]. The health checkup population was mainly from the urban citizens and the dental and TMD examination was just one part of the regular health examination, so we could exclude the sample including bias and the sample could partly reflect the urban general residents taking consideration of the large adult sample. Congenitally missing teeth are more common in female population, but gender variable is still significant for TMD after adjustment, which agrees with previous reports of woman’s vulnerability to TMD [24, 29]. Age is negatively associated with TMD in the adjusted model, similar with previous literature [24]. In the univariate analysis, orthodontic treatment is significant with TMD but orthodontic treatment is not significant with TMD in the adjusted model, indicating there may be no association between orthodontic treatment and TMD [30].

In the present study, we found presence of congenitally missing teeth is a risk factor for TMD including intra-articular TMD and pain-related TMD after adjusting confounders. Genetically modulated TMJ OA models have showed the genetical factors are involved in TMD [25]. The hypodontia may be related to WNT10A polymorphism both in Chinese [31, 32] and western [33] population but WNT10A could also clear senescent synovial resident stem cells and protect cartilage integrity in knee OA joints [34]. In contrary, fibroblast growth factor receptor 1 (FGFR1) mutation is also associated with premolar agenesis [35] but FGFR1 loss inhibits TMJ osteoarthritis [36]. The genetic mechanism linking congenitally missing teeth and TMD progression deserves further studies.

Congenitally missing teeth lead to numerous malocclusion changes due to the Bolton index discrepancy and abnormal craniofacial morphology [7, 15]. These could be indirectly reflected by the significantly higher orthodontic treatment in the congenitally missing teeth group. Although the relationship between malocclusion and TMD is controversial [19], congenitally absent anterior teeth account for 91.60% of the congenitally missing teeth population and often result in deep overjet and overbite, anterior crossbite, asymmetrical malocclusion, abnormal anterior tooth guide and even RCP-ICP slides which are reported to be the risk factors of TMD [21]. Interestingly, although the association of tooth loss and TMD had different literature results [17, 21, 22], we found the number of dental quadrants with missing teeth was significantly associated with overall TMD while the numbers of non-congenitally or congenitally missing teeth were not associated with TMD in the adjusted model even though the number of congenitally missing teeth was significant in the univariate analysis. These results agree with previous reports [23, 24]. In contrary to secondary tooth missing in adults, congenitally missing teeth accompany with occlusion development and may result in drifting and tipping due to the spaces left by the congenitally missing teeth because of the long-term influences and the fast bone turnover in adolescence. In the present study, hypodontia is the majority. Even a small number of congenitally missing teeth was significantly with TMD, this could be partly explained by the so-called “tightly locked occlusion” due to a small number of tooth loss [23], which may impose a different biomechanical effect on TMJ. Although the overall TMD prevalence rates in the congenitally missing posterior teeth group and the group with both congenitally missing anterior and posterior teeth were insignificantly greater than the congenitally missing anterior teeth group, the differences of the overall TMD prevalence among the three congenitally missing teeth groups could not be analyzed further due to the low sample numbers of the congenitally missing posterior teeth group and the group with both congenitally missing anterior and posterior teeth. For the same reason it could not compare the TMD prevalence differences between hypodontia and oligodontia group. Further greater sample number studies may be needed to validate the TMD prevalence differences between hypodontia and oligodontia group or among different congenitally missing sextants.

The limitations of the present study should be discussed. First, the TMD diagnosis was based on clinical examination of symptoms and signs, TMJ images such as CBCT or MRI were not taken into consideration. Second, psychological factors were reported to be associated with pain sensitivity and TMD [26] and information about social/psychological factors should have been added in this present large sample analysis. However, the hypodontia is the majority of our sample and literature has shown hypodontia has limited or no obvious psychosocial impact [37, 38]. The above factors should be kept in mind when one considers the results.

In summary, congenitally missing tooth is a risk factor for TMD. When treating the congenitally missing teeth population, temporomandibular joint evaluation and multidisciplinary strategies are necessary.

## Declarations

### Data statement

The datasets generated and/or analysed during the current study are not publicly available due to potential privacy concerns but are available from the corresponding author on reasonable request.
